# Neuroprotective Potential of Synaptamide in MPTP-Induced Parkinson’s Disease

**DOI:** 10.3390/pathophysiology33030042

**Published:** 2026-06-25

**Authors:** Igor Manzhulo, Yuliya Kipryushina, Ekaterina Gromova, Olga Manzhulo, Elena Milkina, Darya Ivashkevich

**Affiliations:** A.V. Zhirmunsky National Scientific Center of Marine Biology, Far Eastern Branch, Russian Academy of Sciences, Palchevskogo Str., 17, Vladivostok 690041, Russia; yulia.kipryushina@gmail.com (Y.K.); gromova.eser@dvfu.ru (E.G.); olga.manzhulo@bk.ru (O.M.); lotlorien27@gmail.com (E.M.); owncean@yandex.ru (D.I.)

**Keywords:** synaptamide, DHEA, Parkinson’s disease, MPTP, neuroprotection, α-synuclein, neuroinflammation

## Abstract

Background/Objectives. Parkinson’s disease (PD) is a multifactorial neurodegenerative disorder characterized by dopaminergic neuron loss, α-synuclein pathology, neuroinflammation, and cognitive decline. Synaptamide (N-Docosahexaenoylethanolamine (DHEA)) is an endogenous lipid mediator with documented anti-inflammatory and neurogenic properties, but its effects in PD models remain unexplored. This study aimed to evaluate the neuroprotective potential of synaptamide in a subchronic MPTP-induced mouse model of PD. Methods. Male C57BL/6 mice received MPTP (30 mg/kg/day, i.p., 5 days) with or without synaptamide (10 mg/kg/day, s.c., 13 days). Behavioral tests (open field, Y-maze, elevated plus maze, novel object recognition (NOR)) were performed, followed by immunohistochemical analysis of tyrosine hydroxylase (TH)-positive neurons in the substantia nigra, and Western blotting for α-synuclein, p-α-synuclein, TH, and IL1β in brain homogenates and serum. In vitro Neuro-2a cells were co-treated with MPP^+^ (100 µM) and synaptamide (0.1–10 µM) for cytotoxicity assessment (MTS assay). Results. Synaptamide (10 µM) significantly attenuated MPP^+^-induced cytotoxicity in Neuro-2a cells. In vivo, MPTP caused a marked loss of TH^+^-neurons in the substantia nigra, which was prevented by synaptamide treatment. Importantly, this subchronic MPTP model recapitulates early biochemical alterations (e.g., α-synuclein phosphorylation at Ser129) rather than mature Lewy body pathology, a limitation that should be considered when interpreting these findings. Although no motor deficits or anxiety-like behavior were observed, the NOR test revealed MPTP-induced long-term memory impairment, which was fully restored by synaptamide. Conclusions. These findings suggest that synaptamide may exert effects on pathological processes associated with PD, warranting further investigation into its potential role in combination or supportive therapy for this disease.

## 1. Introduction

Parkinson’s disease (PD) is the second most common neurodegenerative disorder after Alzheimer’s disease, demonstrating the highest growth rates of incidence among all neurodegenerative pathologies [1]. The clinical picture of PD consists of motor symptoms, including bradykinesia, muscle rigidity, resting tremor, postural instability and gait disturbances, as well as a wide range of non-motor manifestations, such as cognitive impairment, affective disorders, autonomic dysfunction, constipation, and hyposmia [2,3]. From a pathophysiological perspective, PD is traditionally characterized by progressive and selective loss of dopaminergic neurons in the pars compacta of the substantia nigra, which leads to dopamine depletion in the striatum and serves as the direct cause of the main motor symptoms [4,5]. Alongside neurodegeneration, a key pathological feature of PD is the accumulation of intracellular cytoplasmic protein aggregates known as Lewy bodies, the main component of which is the protein α-synuclein [2,6]. The pathological transformation of α-synuclein involves conformational changes that promote its oligomerization and fibril formation, leading to toxic effects mediated by disruption of cellular homeostasis [7]. Current data, however, present PD as a multifactorial and systemic disease whose pathogenesis extends far beyond isolated dopaminergic dysfunction and α-synuclein aggregation [1]. Mechanisms such as neuroinflammation, mitochondrial dysfunction, protein metabolism impairment, glymphatic system dysfunction, and disturbances in lipid and calcium homeostasis are involved in the development of the pathological process [1,4]. Genetic risk factors, such as mutations in the LRRK2 and GBA1 genes, are associated with both familial and sporadic cases of PD, underscoring the complexity and heterogeneity of the disease mechanisms [2].

Experimental models based on neurotoxins that induce selective degeneration of dopaminergic neurons are widely used for preclinical study of PD pathogenic mechanisms and evaluation of potential neuroprotective compounds. Among the most common toxic models, a special place is occupied by the administration of the compound 1-methyl-4-phenyl-1,2,3,6-tetrahydropyridine (MPTP), which is capable of crossing the blood–brain barrier and, after biotransformation into the active metabolite MPP^+^ (1-methyl-4-phenylpyridinium) by monoamine oxidase B in glial cells, selectively binds to mitochondrial complex I of the respiratory chain, causing the generation of reactive oxygen species, mitochondrial dysfunction, and subsequent selective death of dopaminergic neurons in the substantia nigra [3,5]. The MPTP-induced mouse model remains the most frequently used in preclinical studies due to its reproducibility, relatively low cost, and ability to cause specific damage to the nigrostriatal pathway identical to that observed in PD [8,9]. Different MPTP administration regimens (acute, subchronic, and chronic) allow modeling of various stages and aspects of the pathological process [10]. The subchronic regimen, involving daily MPTP administration for 5 days, induces a pronounced loss of tyrosine hydroxylase-positive neurons and activation of neuroinflammatory processes in the substantia nigra of C57BL/6 mice [11,12]. Importantly, this subchronic MPTP regimen in C57BL/6 mice does not typically produce overt motor deficits because the degree of dopamine loss (typically 40–50%) remains below the threshold (approximately 80%) required for detectable motor impairments [12]. This feature makes the model particularly suitable for studying early biochemical and cognitive alterations before the onset of severe motor dysfunction. However, unlike chronic or genetic models, this subchronic MPTP regimen does not lead to the formation of mature Lewy bodies; rather, it induces early, reversible phosphorylation of α-synuclein at Ser129 [12].

In the context of searching for new neuroprotective compounds capable of targeting key pathogenic mechanisms of PD, endogenous lipid mediators are of particular interest. Synaptamide (N-docosahexaenoylethanolamine, DHEA) is an endogenous metabolite of docosahexaenoic acid (DHA, 22:6n-3)—an omega-3 polyunsaturated fatty acid that is present in high concentrations in brain tissue and plays an important role in neuronal development, synaptic plasticity, and neuroprotection [13]. The key mechanism mediating the neuroprotective and anti-inflammatory effects of synaptamide is its binding to the G protein-coupled receptor GPR110 (ADGRF1). Activation of GPR110 leads to the accumulation of intracellular cyclic adenosine monophosphate (cAMP), subsequent activation of protein kinase A (PKA), and phosphorylation of the transcription factor CREB, which promotes the expression of neurogenic and synaptogenic genes as well as the suppression of pro-inflammatory gene expression [13]. In preclinical models of neuropathology, including a mild traumatic brain injury model, subcutaneous administration of synaptamide at a dose of 10 mg/kg demonstrated significant anti-neuroinflammatory and anti-apoptotic effects [14,15]. Synaptamide reduced the expression of pro-inflammatory cytokines IL1β, IL6, and TNFα in the cerebral cortex, promoted M2 polarization of microglia, suppressed the expression of pro-apoptotic proteins Bad and Bax, and increased the expression of the anti-apoptotic protein Bcl-2, which was accompanied by an improvement in the morphological state of neurons and restoration of cognitive functions in the Y-maze test [14,15]. In a traumatic brain injury model, synaptamide also restored the production of the synaptic plasticity-associated protein Arc/Arg3.1 and increased the number of Ki-67-positive proliferating cells and DCX-positive newly generated neurons in the subgranular zone of the hippocampal dentate gyrus [15]. Accumulated evidence suggests that synaptamide can effectively modulate the pathogenetic mechanisms that play a central role in the development and progression of PD: neuroinflammation, oxidative stress, apoptosis, and impaired synaptic plasticity. However, despite compelling evidence of synaptamide’s neuroprotective activity in various neuropathological conditions, its effect on the MPTP-induced model of PD remains unexplored.

The aim of this study is to evaluate the neuroprotective potential of synaptamide administered subcutaneously at a dose of 10 mg/kg in C57BL/6 mice with a subchronic MPTP-induced model of PD, with the goal of identifying preliminary correlations between synaptamide treatment and key pathological markers.

## 2. Materials and Methods

### 2.1. Synaptamide (N-Docosahexaenoylethanolamine, DHEA)

The synthesis of synaptamide (purity 99.4%) was carried out at NSCMB FEB RAS via chemical modification of docosahexaenoic acid extracted from the digestive gland of Berryteuthis magister. The detailed synthetic route has been described earlier [14]. For in vivo administration, synaptamide was formulated as an ethanol-stabilized emulsion, diluted with sterile water right before use, and thoroughly mixed on a vortex shaker (BioSan, Multi-Vortex V-32, Riga, Latvia). Subcutaneous injections were given daily for 13 consecutive days at a dose of 10 mg/kg in a volume of 100 µL per animal. Control groups (“Veh” and “MPTP”) received an equivalent volume of vehicle solution (sterile water containing 1.5% ethanol).

### 2.2. PD Modeling

**Figure 1:**
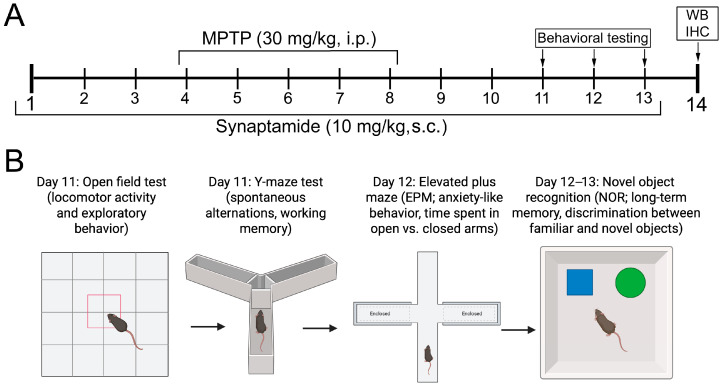
(**A**) Timeline of the experiment. Synaptamide (10 mg/kg, s.c.) or vehicle was administered daily from day 1 to day 13. MPTP (30 mg/kg, i.p.) or water was administered daily from day 4 to day 8 (5 days). (**B**) Behavioral tests were conducted on days 11–13: open field, Y-maze, elevated plus maze (EPM), and novel object recognition (NOR).

For the experimental model, an administration regimen of the inducer MPTP (1-methyl-4-phenyl-1,2,3,6-tetrahydropyridine, Macklin, Shanghai, China) used for modeling PD was selected. A detailed scheme of drug administration and in vivo experiments is shown in Figure 1. Three days before intraperitoneal administration of MPTP (30 mg/kg/5 days), synaptamide (10 mg/kg/13 days) was administered subcutaneously and its administration continued until day 14 of the experiment. On days 11–13 of the experiment, physiological testing of the animals was performed; on day 14, the mice were sacrificed. All experiments involved three-month-old male C57BL/6 mice weighing 28–30 g, housed under standard conditions (temperature 23 ± 2 °C, humidity 55 ± 15%, 12 h light/dark cycle) with food and water provided ad libitum. Animals were kept in groups of 5–6 individuals per cage and had unlimited access to water and standard rodent chow. A total of 44 animals were used in the experiment, with 11 mice in each group: “Veh”—mice with subcutaneous administration of synaptamide vehicle and intraperitoneal administration of water; “Veh+Syn”—mice with subcutaneous administration of synaptamide and intraperitoneal administration of water; “MPTP”—mice with subcutaneous administration of synaptamide vehicle and intraperitoneal administration of MPTP; “MPTP+Syn”—mice with subcutaneous administration of synaptamide and intraperitoneal administration of MPTP. All procedures received prior approval from the Animal Ethics Committee at the A.V. Zhirmunsky National Scientific Center of Marine Biology, Far Eastern Branch, Russian Academy of Sciences (Protocol No. 32, 31 March 2026), following the European Directive 2010/63/EU.

### 2.3. Behavioral Tests

To evaluate locomotor and cognitive functions, we performed a series of behavioral tests in the following order: open field (locomotor activity), Y-maze (working memory), elevated plus maze (anxiety-like behavior), and novel object recognition (long-term memory). Testing was carried out over three consecutive days (days 11–13) to avoid any confounding effects of excessive handling.

#### 2.3.1. Open Field Test

Locomotor activity was assessed using a square arena (40 cm × 40 cm) made of opaque plastic with 40 cm-high walls. Each mouse was placed in the center of the arena, and its movements were video-recorded for 5 min. Between trials, the arena was cleaned with 20% ethanol to eliminate olfactory cues. The floor was divided into 8 cm × 8 cm squares, and locomotor activity was quantified as the total number of line crossings (defined as entry of all four paws or at least both front paws into a new square).

#### 2.3.2. Novel Object Recognition Test (NOR)

Hippocampus-dependent long-term memory was assessed using the NOR test, which comprised three phases. On the first day (training), two identical unfamiliar objects (wooden cylinders) were introduced, and the animal was allowed to explore them for 5 min. Following 24 h interval (testing phase), one of the familiar objects was replaced with a novel one (differing in shape, color, and texture—a triangle), and exploration was recorded for another 5 min. The time spent actively exploring each object (nose directed toward the object at a distance ≤ 2 cm or touching it) was measured in seconds.

During the test phase, the discrimination index (DI) was calculated as:

DI = (T_C_ − T_A_)/(T_C_ + T_A_)

where T_A_ is the time spent exploring the familiar object (A) and T_C_ is the time spent exploring the novel object (C). The DI ranges from −1 to +1, with positive values indicating a preference for the novel object (intact long-term memory), values near zero indicating no discrimination (memory impairment), and negative values indicating a preference for the familiar object.

#### 2.3.3. Y-Maze Test

Working spatial memory was evaluated using a Y-shaped maze constructed from opaque plastic, with three arms (each 35 cm long, 8 cm wide, 15 cm high) positioned at 120° angles. Each mouse was placed in the center and allowed to explore freely for 5 min. Arm entry was counted when all four paws were placed entirely inside an arm. The percentage of spontaneous alternations was calculated as the number of triads consisting of entries into three different arms divided by the total possible alternations (N–2), multiplied by 100%.

#### 2.3.4. Elevated Plus Maze (EPM)

Anxiety-like behavior was measured using the elevated plus maze, which consisted of two open arms (30 cm × 5 cm, 0.25 cm walls), two enclosed arms (30 cm × 5 cm × 15 cm), and a central open platform (5 cm × 5 cm), elevated above the floor. Illumination levels were approximately 150 lux in the open arms and 5 lux in the enclosed arms. Each animal was positioned on the central platform facing an enclosed arm, and behavior was recorded for 5 min. Parameters analyzed included time spent in open arms, closed arms, and the central platform, using automated tracking software (SMART 3.0, Panlab/Harvard Apparatus, Holliston, MA, USA).

### 2.4. Immunohistochemical Study

Tissue harvesting for immunohistochemistry was performed on day 14. Mice (n = 7 per group) were deeply anesthetized with 3% isoflurane in 100% oxygen using a rodent vaporizer (VetFloTM, Kent Scientific Corporation, Torrington, CT, USA), followed by transcardial perfusion with ice-cold 0.1 M phosphate-buffered saline (PBS, pH 7.2) and then with 10% buffered formalin. Brains were removed, post-fixed in the same fixative for 24 h at 4 °C, washed five times with PBS, dehydrated, and embedded in paraffin. For Western blotting, blood serum and brain tissues (n = 4 per group) were collected without perfusion, snap-frozen in liquid nitrogen, and stored at −80 °C.

Brain sections (7 µm thick) were deparaffinized, treated with 3% hydrogen peroxide for 15 min to block endogenous peroxidase, and rinsed three times with PBS. Sections were then incubated for 1 h in blocking solution (PBS containing 2% bovine serum albumin (BSA, SC-2323, Santa Cruz Biotechnology, Dallas, CA, USA), 0.1% Tween20, and 0.25% Triton X-100 (Sigma, St. Louis, MO, USA)). Primary polyclonal rabbit anti-TH antibody (1:500, Vector Laboratories T 489, Newark, CA, USA) diluted in blocking buffer was applied overnight at 4 °C in a humidified chamber. Negative controls were incubated without primary antibody. After three PBS washes, sections were incubated with biotinylated goat anti-rabbit secondary antibody (ab178846, Abcam, Waltham, MA, USA) for 15 min, followed by streptavidin (ab64269, Abcam, Waltham, MA, USA) for 10 min, with intermediate PBS washes. Peroxidase activity was visualized using DAB chromogen (ab64238, Abcam, Waltham, MA, USA) for 5 min. Sections were then washed, dehydrated, and coverslipped with mounting medium (CS705, Dako, Carpinteria, CA, USA).

The number of TH-immunopositive cells in the substantia nigra was calculated using the formula:

D = (10^9^ × n)/(S × 7), where D is cell density (cells/mm^3^), 10^9^ is the conversion factor from µm^2^ to mm^3^, n is the number of positive cells, S is the area of the region of interest (µm^2^), and 7 is the section thickness (µm).

### 2.5. Western Blotting

Brain tissues were manually homogenized using a pestle in PBS (0.1 M, pH 7.2) containing 150 mM PMSF. Blood serum of experimental animals was thawed and diluted with 150 mM PMSF in PBS (0.1 M, pH 7.2), without homogenization. The protein concentration in samples was adjusted to 2 mg/mL. Subsequently, loading buffer (Sample Buffer, Biorad, Hercules, CA, USA) supplemented with 5% 2-mercaptoethanol (Sigma-Aldrich, M6250, St. Louis, MO, USA) was added to the samples in a 1:1 ratio. Then the samples were incubated at 94 °C in a water bath for 5 min. For electrophoretic analysis, ready-made 4–15% gel cartridges (Macklin, Shanghai, China) and Spectra Multicolor Broad Range Protein Ladder (Thermo Fisher, Waltham, MA, USA) were used in a Biorad chamber. For the analysis of markers in blood serum, brain substantia nigra, and loading control, the same loading of 40 mg/well was used. Subsequently, a Vertical Mini Trans-blot Electrophoretic Transfer Cell (Biorad, Hercules, CA, USA) was used to transfer samples to PVDF membranes. Transfer was performed for 1 h, voltage was 120 V, current was 350 mA. After transfer, membranes were stained with Ponceau S for additional loading control. Then the membranes were washed from the stain using PBS (0.1 M, pH 7.2) containing 0.1% Tween 20 (PBS-T). Next, membranes were incubated overnight in blocking buffer (0.1 M PBS, pH 7.2, with 2% BSA). The next day, membranes were placed overnight in primary antibody solution at 4 °C, after preliminary washing of membranes 3 times for 10 min in PBS-T. Primary antibodies used included α-tubulin (1:1000, AF0524, Affinit, Shanghai, China), α-syn (106209-T08, Sino Biological, Beijing, China), p-α-syn (1:1000, AF3285, Affinit, Shanghai, China), TH (1:1000, Vector Laboratories T 489, Newark, CA, USA), IL1β (1:1100, BF8021, Affinit, Shanghai, China), diluted in PBS-T. Due to overlapping molecular weights, each marker was run on a separate gel with its corresponding loading control. Membranes were then washed three times with PBS-T and incubated with HRP-conjugated secondary antibodies (anti-mouse or anti-rabbit, Vector Laboratories, 1:2000) for 1 h. After three final washes, immunoreactive bands were visualized using chemiluminescent ECL substrate (Biorad, Hercules, CA, USA) and imaged with a ChemiDoc system (Biorad, Hercules, CA, USA). Band intensities were quantified using ImageJ 1.41 (NIH, Bethesda, MD, USA).

### 2.6. Cell Culture

The mouse neuroblastoma Neuro-2a cell line (CCL-131, ATCC, Manassas, VA, USA) was maintained in High Glucose DMEM (4.5 g/L glucose) supplemented with 10% fetal bovine serum and 0.5% penicillin/streptomycin at 37 °C in a humidified atmosphere containing 5% CO_2_. Cells were passaged using 0.05% trypsin-EDTA. All cell culture reagents were obtained from Thermo Fisher Scientific (Waltham, MA, USA).

### 2.7. Cytotoxicity Assay

To assess the cytoprotective activity of synaptamide, Neuro-2a cells were seeded at 1 × 10^5^ cells/cm^2^ in 96-well plates and allowed to adhere for 24 h. The cells were then co-treated with MPP^+^ (100 µM, M875357, Macklin, Shanghai, China) and increasing concentrations of synaptamide (0.1, 1, and 10 µM) for 24 h. Negative controls received fresh medium only, while positive controls were exposed to MPP^+^ alone. After the incubation period, MTS reagent (ab197010, Abcam, Waltham, MA, USA) was added to each well, and the plates were incubated for an additional 2 h at 37 °C. Absorbance was measured at 490 nm using a microplate reader (Biorad, Hercules, CA, USA). Cell viability was expressed as a percentage relative to the negative control.

### 2.8. Statistical Analysis

All statistical analyses and graphical representations were performed using GraphPad Prism 8.00 (GraphPad Software, San Diego, CA, USA). Normality was checked with the Shapiro–Wilk test. For experiments with two independent categorical variables, two-way ANOVA followed by Tukey’s post hoc test was used. For the DI in the NOR test phase, two-way ANOVA was followed by Fisher’s Least Significant Difference (LSD) post hoc test, as this test is appropriate for planned comparisons when the overall ANOVA indicates significant main effects without a significant interaction. For the NOR test, comparisons of exploration time between the familiar and novel objects within each experimental group were performed using Student’s *t*-test. For the in vitro MTS assay, one-way ANOVA followed by Tukey’s post hoc test was used. Data are presented as mean ± SEM (standard error of the mean), and values of *p* < 0.05, *p* < 0.01, *p* < 0.001 were considered statistically significant.

## 3. Results

### 3.1. Neuroprotective Effect of Synaptamide In Vitro

**Figure 2:**
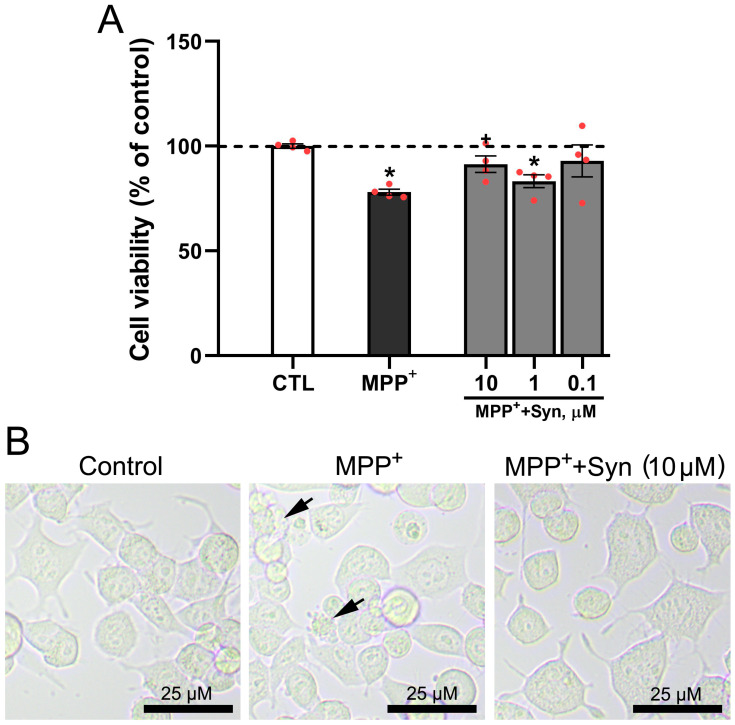
(**A**) Evaluation of the neuroprotective effect of synaptamide (0.1–10 µM) upon co-cultivation of Neuro-2a neuroblasts with MPP^+^. Data are mean ± SEM, n = 4 (independent experiments), * *p* < 0.05 compared to “CTL”, ^+^ *p* < 0.05 compared to “MPP^+^” (MTS test) (one-way ANOVA followed by Tukey’s post hoc test). (**B**) Representative phase-contrast images showing morphological changes. Rounded cells detached from the substrate (black arrows).

The MTS assay revealed that treatment of Neuro-2a neuroblasts with MPP^+^ at a concentration of 100 µM caused a significant decrease in cell viability (by 25% compared to control). Co-addition of MPP^+^ and synaptamide to the culture medium was accompanied by neuroprotective activity of synaptamide at a concentration of 10 µM (Figure 2A). Morphological changes in cells showed a similar trend. In the control group, cells were polygonal, elongated, tightly attached to the substrate and to each other, the cytoplasm was relatively homogeneous, cell contours were distinct; nuclei were not prominent. There were no large vacuoles, membrane blebbing, or significant rounding; overall, the morphology was characteristic of a viable adherent neuroblast culture. Upon addition of MPP^+^ to the cells, the following pathomorphological phenomena were observed in the culture: clear loss of adhesion in some cells; many cells were rounded, partially or completely detached from the substrate (black arrows). Large intracellular clear cavities in the cytoplasm of several cells were visible in the field of view, indicating pronounced vacuolization. Some cells appeared shrunken and/or with denser (darker) nuclei, which may indicate nuclear pyknosis or apoptotic changes. Small structures resembling apoptotic bodies were visible in places within the field of view. The altered cell morphology indicated pronounced cellular stress and MPP^+^-induced cytotoxicity. When synaptamide (10 µM) was added to the medium, the cell condition significantly improved: adhesion to the substrate was preserved, fewer rounded cells were observed, the cytoplasm appeared more homogeneous, vacuolization was less pronounced or absent. Nuclear and cellular contours were clearer, and there were fewer obvious apoptotic bodies (Figure 2B). Thus, synaptamide likely exhibits a cytoprotective effect and reduces the phenotypic signs of MPP^+^-induced stress. These morphological observations are presented as qualitative illustrations of the cellular state, complementing the quantitative MTS viability data.

### 3.2. Restoration of Long-Term Memory by Synaptamide in the PD Model

**Figure 3:**
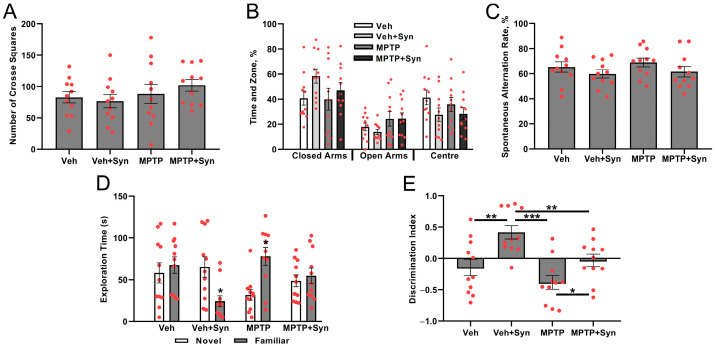
Physiological testing of animals in the PD model and upon synaptamide administration. (**A**) Open field test, graph shows the average number of square crossings. (**B**) Elevated plus maze test, graph shows the average time spent in open and closed arms, as well as on the central platform. (**C**) Y-maze test, graph shows the percentage of spontaneous alternations. (**D**) Novel object recognition test, graph evaluates the average time spent by the animal with the new and old object. (**E**) The graph evaluates the discrimination index in the NOR test. Data are mean ± SEM, n = 11 (animals/group), * *p* < 0.05, ** *p* < 0.01, *** *p* < 0.001 ((**D**) Student’s *t*-test; (**E**) two-way ANOVA followed by post hoc Fisher’s LSD test).

In the open field test, no significant differences in the number of squares crossed were found between experimental groups (Figure 3A). In the elevated plus maze test, no statistically significant differences between groups were also revealed; however, a tendency towards increased time spent in the closed arms and decreased time on the central platform was observed both in animals receiving synaptamide and in animals with MPTP-induced PD (Figure 3B). In the spontaneous alternation test (Y-maze), no significant differences between groups were recorded either, although a certain tendency towards an increase in this parameter was noted in animals receiving MPTP (Figure 3C). Consistent with these observations, two-way ANOVA revealed no significant main effects or interactions for any of the parameters measured in the open field, Y-maze, or elevated plus maze tests. This absence of motor deficits is consistent with previous characterizations of the subchronic MPTP model in C57BL/6 mice, where striatal dopamine loss remains below the threshold for detectable motor abnormalities [12]. Therefore, the selected model does not produce pronounced motor impairments, anxiety, or working memory deficits, which is not a limitation but a well-documented feature of this specific regimen, allowing focused investigation of cognitive and biochemical endpoints without confounding severe motor disability. In the novel object recognition (NOR) test, control animals demonstrated approximately equal time exploring the old and new objects, indicating the absence of spontaneous memory impairments. Control animals receiving synaptamide spent significantly more time with the new object (63% more), which may reflect a cognitive stimulatory effect of the compound. In the “MPTP” group, the time spent with the new object was 60% less than with the old object, indicating long-term memory impairment. In the “MPTP+Syn” group, restoration of this parameter to control values was observed (Figure 3D). During the test phase, the discrimination index (DI) was calculated as a measure of long-term memory retention. Two-way ANOVA of the DI revealed a significant main effect of synaptamide treatment (F(1,40) = 15.48; *p* = 0.0003), a significant main effect of MPTP treatment (F(1,40) = 11.60; *p* = 0.0015), and no significant interaction between the two factors (Figure 3E). Post hoc comparisons using Fisher’s LSD test showed that the DI was significantly lower in the “MPTP” group compared to the “Veh+Syn” group (*p* < 0.001) and compared to the “MPTP+Syn” group (*p* = 0.0278). The DI was significantly higher in the “Veh+Syn” group compared to the “Veh” group (*p* = 0.0021) and compared to the “MPTP+Syn” group (*p* = 0.0059). No significant difference was observed between the “Veh” and “MPTP” groups (*p* = 0.0635) nor between the “Veh” and “MPTP+Syn” groups (*p* = 0.7101). Taken together, these data indicate that the subchronic MPTP model selectively impairs hippocampal-dependent long-term memory, as reflected by a negative discrimination index in the “MPTP” group. Synaptamide treatment partially ameliorated this deficit, as evidenced by a higher DI in the “MPTP+Syn” group compared to the “MPTP” group (*p* = 0.0278). However, the DI in the “MPTP+Syn” group did not fully reach the level observed in the “Veh+Syn” group, indicating that while synaptamide improves memory performance, it does not completely restore it to the level seen in healthy animals treated with the compound alone.

### 3.3. Synaptamide Attenuates the MPTP-Induced Increase in Serum p-α-syn and IL1β Levels as Biomarkers of PD

**Figure 4:**
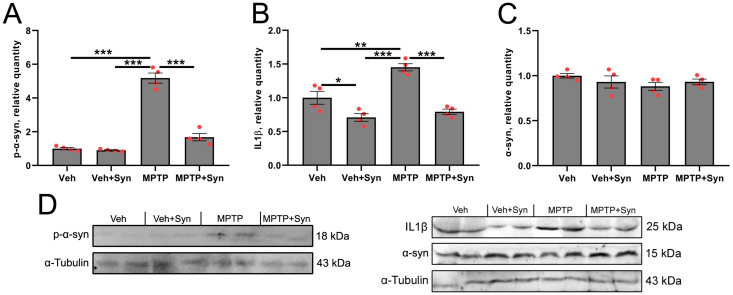
Western blot analysis of (**A**) p-α-syn, (**B**) IL1β, (**C**) α-syn expression in the blood serum of experimental animals. Expression levels were normalized to α-Tubulin and expressed relative to control (“Veh” group). Data are mean ± SEM, n = 4 animals/group. * *p* < 0.05, ** *p* < 0.01, *** *p* < 0.001 (two-way ANOVA followed by Tukey’s post hoc test). (**D**) Representative immunoblots for each marker (Original images can be found in Supplementary Materials).

Analysis of blood serum of experimental mice revealed a multiple increase in the level of phosphorylated α-synuclein (p-α-syn) upon MPTP administration (Figure 4A,D), suggesting a systemic response to nigrostriatal injury, although the precise origin of this p-α-syn (CNS-derived versus peripheral sources such as blood cells or autonomic nerves) cannot be determined from serum measurements alone. The level of the pro-inflammatory cytokine IL1β was also elevated compared to the control level (Figure 4B,D). Synaptamide administration was accompanied by a reduction in p-α-syn and IL1β levels to control values (Figure 4A,B,D). At the same time, no change in the level of non-phosphorylated (normal) α-syn was observed in all experimental groups (Figure 4C,D). In healthy animals receiving synaptamide, the levels of all studied proteins did not differ from control values. Two-way ANOVA confirmed significant main effects of MPTP treatment (F(1,12) = 172.4; *p* < 0.0001), synaptamide treatment (F(1,12) = 91.08; *p* < 0.0001), and a significant interaction between the two factors (F(1,12) = 81.86; *p* < 0.001) for serum p-α-syn levels. For serum IL1β, two-way ANOVA revealed significant main effects of MPTP treatment (F(1,12) = 17.31; *p* = 0.0013), synaptamide treatment (F(1,12) = 54.28; *p* < 0.0001), and a significant interaction (F(1,12) = 8.120; *p* = 0.0146). For serum total α-syn, two-way ANOVA showed a significant main effect of MPTP treatment (F(1,12) = 9.334; *p* = 0.01), a significant interaction between MPTP and synaptamide (F(1,12) = 9.365; *p* = 0.0099), but no significant main effect of synaptamide treatment alone (F(1,12) = 1.778; *p* = 0.2072). The obtained results indicate that MPTP administration is associated with elevated serum levels of p-α-syn and the pro-inflammatory cytokine IL1β. While these changes may reflect systemic inflammatory and α-synuclein phosphorylation responses to nigrostriatal damage, serum measurements alone cannot distinguish between CNS-derived and peripherally derived p-α-syn. Therefore, these findings are best interpreted as peripheral biomarker changes rather than direct evidence of CNS α-synuclein pathology. Synaptamide effectively reduces both responses, although whether this corresponds to prevention of Lewy body-like pathology remains unknown, as such aggregates do not typically form in this model PD.

### 3.4. Synaptamide Prevents the Increase in p-α-syn Expression in the Substantia Nigra of the Brain upon MPTP Administration

**Figure 5:**
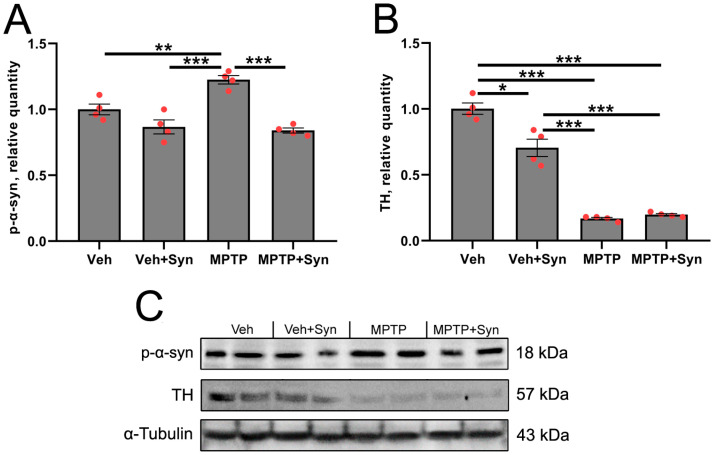
Western blot analysis of (**A**) p-α-syn, (**B**) TH expression in the substantia nigra of the brain of experimental animals. Expression levels were normalized to α-Tubulin and expressed relative to control (“Veh” group). Data are mean ± SEM, n = 4 (animals/group), * *p* < 0.05, ** *p* < 0.01, *** *p* < 0.001 (two-way ANOVA followed by Tukey’s post hoc test). (**C**) Representative immunoblots for each marker (Original images can be found in Supplementary Materials).

Western blot analysis of substantia nigra homogenates brain tissue directly affected by MPTP-induced neurodegeneration revealed similar dynamics in the changes of pathological protein levels. Unlike serum measurements (Section 3.3), tissue analysis allows a more direct assessment of CNS α-synuclein phosphorylation. In the “MPTP” group, a significant increase in the level of p-α-syn compared to control. Synaptamide administration completely normalized the level p-α-syn to control values (Figure 5A,C). Simultaneously, the level of tyrosine hydroxylase (TH) in the substantia nigra in the “MPTP” group was significantly reduced relative to control values. However, synaptamide therapy did not lead to a significant increase in TH levels (Figure 5B,C). In healthy animals receiving synaptamide, the levels of all studied proteins did not differ from control values. Two-way ANOVA of substantia nigra homogenates revealed significant main effects and interactions for all markers analyzed. For p-α-syn, significant effects were observed for MPTP treatment (F(1,12) = 6.670; *p* = 0.0240), synaptamide treatment (F(1,12) = 45.80; *p* < 0.0001), and their interaction (F(1,12) = 10.90; *p* = 0.0063). For TH, two-way ANOVA revealed significant main effects of MPTP treatment (F(1,12) = 287.1; *p* < 0.0001), synaptamide treatment (F(1,12) = 11.40; *p* = 0.0055), and a significant interaction (F(1,12) = 17.09; *p* = 0.0014). The significant interaction between MPTP and synaptamide treatment for all markers in the substantia nigra indicates that the effect of synaptamide is conditional on MPTP-induced pathology, rather than reflecting a non-specific reduction of baseline protein levels. Thus, in the substantia nigra, MPTP induces increased elevated p-α-syn levels, which are early molecular alterations rather than fully developed α-synuclein aggregation.

### 3.5. Synaptamide Is Associated with Preservation of TH-Immunopositive Cell Bodies in the Substantia Nigra Following MPTP Administration

**Figure 6:**
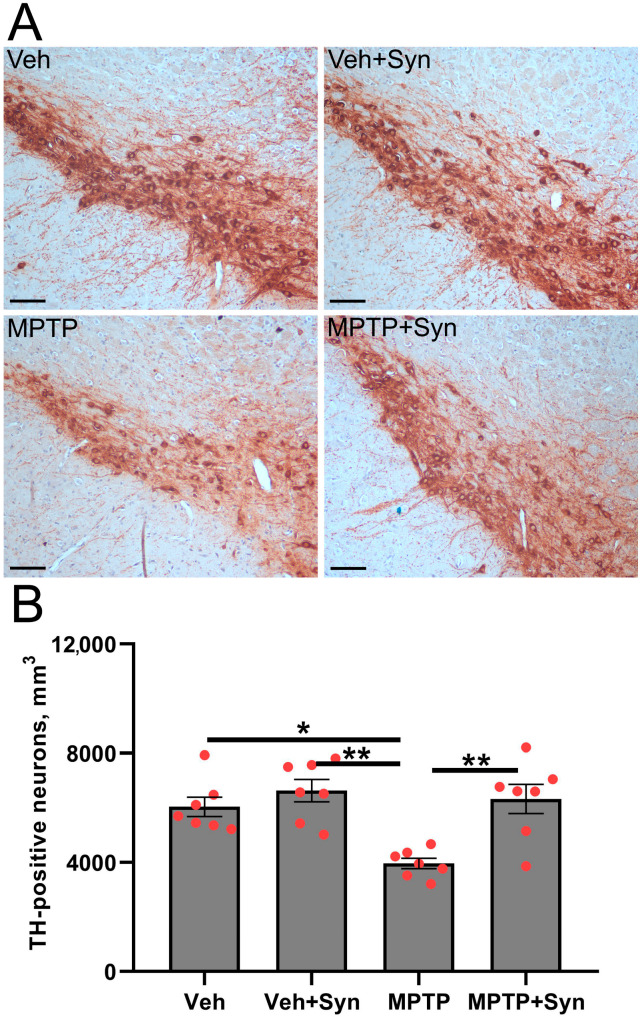
(**A**) Immunoperoxidase mapping of TH-positive neurons in the substantia nigra of the brain of experimental animals. (**B**) Number of TH-positive neurons in the substantia nigra of the brain. Data are mean ± SEM, n = 7 (animals/group). For each animal, multiple substantia nigra sections were analyzed, and the values were averaged to obtain a single biological replicate per animal, * *p* < 0.05, ** *p* < 0.01 (two-way ANOVA followed by Tukey’s post hoc test).

Immunohistochemical examination of the substantia nigra for tyrosine hydroxylase was performed on 7 animals per group, with multiple sections analyzed per animal and the values averaged to obtain a single biological replicate per animal. Two-way ANOVA revealed a significant main effect of MPTP treatment (F(1,24) = 9.24; *p* = 0.0056), a significant main effect of synaptamide treatment (F(1,24) = 14.19; *p* = 0.0009), and a significant interaction between the two factors (F(1,24) = 5.079; *p* = 0.0336). Post hoc comparisons showed a significant decrease in the number of TH-positive neurons in the “MPTP” group (3962 ± 190.9 cells/mm^3^) compared to the “Veh” group (6042 ± 357.4 cells/mm^3^). In animals receiving synaptamide (“MPTP+Syn”), the number of TH-positive neurons remained at control level (6328 ± 532.9 cells/mm^3^). In healthy animals receiving synaptamide (“Veh+Syn”), no significant difference from the “Veh” group was observed (6636 ± 411.9 cells/mm^3^) (Figure 6A,B). The obtained data suggest that synaptamide may protect dopaminergic neurons of the substantia nigra in MPTP-induced damage as assessed by conventional immunohistochemical quantification of TH^+^-cells.

## 4. Discussion

According to current literature data, the pathogenesis of PD extends far beyond the nigrostriatal dopamine system. The key links of the pathological process are aggregation and phosphorylation of α-synuclein, neuroinflammation, tauopathy, mitochondrial dysfunction, and impaired neurogenesis, which form a complex interconnected network leading to neuronal death [16]. In this context, neuroprotective strategies aimed at simultaneously targeting multiple pathogenetic links are considered the most promising approach to developing PD therapy.

In the present study, a subchronic MPTP model in C57BL/6 mice was used, which, according to literature data, is characterized by pronounced neurochemical damage to the dopaminergic system but does not lead to the development of overt motor defects [12,17]. Indeed, in our experiments, the open field, elevated plus maze, and Y-maze tests did not reveal significant differences between groups. This absence of motor deficit and anxiety is not an artifact but represents a normative characteristic of the chosen model, related to the fact that dopamine loss in such a model (40–50%) generally does not reach the threshold necessary for the development of gross motor impairments, which exceeds 80% [12]. Moreover, several studies have shown that subchronic MPTP administration can even cause transient hyperactivity rather than hypoactivity, which may also mask cognitive impairments in tests such as the Y-maze [17]. The only behavioral test that revealed statistically significant differences was the novel object recognition test, which assesses long-term hippocampal-dependent memory. Animals receiving MPTP showed a decreased discrimination index, indicating long-term memory impairment. This result is fully consistent with literature data showing that cognitive deficits in MPTP models more often manifest in tests of long-term rather than working memory [18]. Importantly, the improvement in NOR performance observed in the “MPTP+Syn” group clearly indicates that synaptamide effectively corrects MPTP-induced cognitive deficit. The sensitivity of the NOR test to the therapeutic action of synaptamide is explained by its neuroprotective mechanism of action. MPTP and its toxic metabolite MPP^+^ cause not only damage to the nigrostriatal system but also microgliosis in the hippocampus, which has been demonstrated, in particular, in a chronic model [10] and confirmed in studies with subchronic administration [18]. Damage to the hippocampus, critically important for long-term memory formation, is accompanied by reduced neurogenesis and neuroinflammation. In the work of Joseph and colleagues [18], it was shown that MPTP (30 mg/kg/5 days) reduces the number of doublecortin-positive (DCX) immature neurons and the survival rate of newborn cells in the hippocampal dentate gyrus. Synaptamide is known to improve hippocampal neurogenesis and restore long-term potentiation (LTP), which directly links its therapeutic effect in the NOR test to the restoration of hippocampal neuroplasticity [18,19].

Although the NOR test and hippocampal-dependent changes are the most sensitive markers of therapeutic efficacy in our model, the key pathomorphological substrate of PD remains degeneration of the nigrostriatal system. The significant loss of TH-positive neurons revealed by immunohistochemical analysis of the substantia nigra is consistent with data on pronounced neurochemical damage in this model [20]. The most important observation was that synaptamide therapy prevented this loss, and the number of TH^+^-neurons in the “MPTP+Syn” group did not differ from control values. However, when analyzing the same tissues by Western blotting, no significant increase in TH protein level in brain homogenate was detected, despite obvious neuroprotection at the cellular level. This apparent contradiction can be explained by the fact that immunohistochemistry assesses the number of living, functionally active neurons expressing TH, whereas Western blot measures the total protein pool in the homogenate, which includes protein from damaged and degenerating cells not yet eliminated from the tissue. Since the subchronic model is characterized by transient and partial damage rather than total neuronal death as in the acute regimen [21], the contribution of preserved neurons to the total homogenate may be insufficient for a statistically significant increase in TH by Western blot. However, this discrepancy also raises an important limitation of the present study. First, we did not perform stereological quantification of TH^+^-neurons, which is the gold standard for unbiased cell counting. Our density-based quantification (cells/mm^3^) may overestimate the protective effect due to tissue shrinkage or sampling bias. Second, we did not assess TH fiber density in the striatum this is a functionally relevant parameter, as dopaminergic terminal loss often precedes and predicts perikaryal loss. Third, we did not measure striatal dopamine or its metabolites by HPLC, which would provide direct biochemical evidence of preserved dopaminergic function.

At the molecular level, the protective effect of synaptamide may be mediated through modulation of several key pathophysiological pathways. MPTP administration led to a significant increase in serum p-α-syn levels. While this finding is consistent with previous reports of elevated α-synuclein phosphorylation in MPTP models [20], it is important to note that serum p-α-syn may originate from multiple sources, including peripheral blood cells (which contain abundant α-synuclein), the enteric nervous system, or sympathetic nerves, rather than directly reflecting CNS pathology. Indeed, the relationship between serum and CNS α-synuclein species remains incompletely understood, and serum p-α-syn should not be equated with brain α-synuclein aggregation or Lewy body pathology. Therefore, we interpret the reduction in serum p-α-syn upon synaptamide treatment as a peripheral biomarker response that correlates with CNS protection but does not itself demonstrate direct CNS target engagement. The same applies to serum IL1β, which reflects systemic inflammation and may not precisely mirror neuroinflammation within the substantia nigra or hippocampus. A critical limitation of the subchronic MPTP model used in this study must be explicitly acknowledged before interpreting the p-α-syn data. While MPTP reliably induces nigrostriatal dopaminergic degeneration and increases α-synuclein phosphorylation at Ser129, this model does not recapitulate the formation of mature Lewy bodies or progressive, irreversible α-synuclein aggregation [8,22]. Therefore, our observations of reduced p-α-syn following synaptamide treatment should be interpreted as effects on early, reversible phosphorylation events rather than prevention of aggregate or tangle formation. The translational relevance of these findings to human PD, which involves decades of progressive protein aggregation, remains to be established. According to the mechanism proposed by Qureshi and Paudel, MPTP increases the level of α-synuclein, leading to microtubule destabilization and neuronal death [23,24]. By reducing p-α-syn levels, synaptamide may break this pathological link, preventing pathological activation of GSK-3β [24]. Moreover, it has been shown that other neuroprotective agents also reduced p-α-syn level, indicating the existence of a common protective mechanism, which may also be the case for synaptamide [25,26].

Integrating the obtained data, we hypothesize that synaptamide may contribute to neuroprotection through several potential mechanisms: under conditions of MPTP-induced oxidative stress, activation of kinases such as GSK-3β and phosphorylation of α-synuclein. Elevated p-α-syn levels and neuroinflammation create a toxic environment, especially in the hippocampus, leading to suppression of neurogenesis and impairment of long-term memory. Based on previous reports [27,28,29], synaptamide is likely to cross the blood–brain barrier, where it could modulate p-α-syn levels, suppress neuroinflammation. By reducing p-α-syn level, synaptamide may stabilize synaptic function and reduce the aggregation potential of the protein. The result of these molecular events would be the preservation of dopaminergic neuron survival in the substantia nigra, restoration of neurogenesis in the hippocampus, and, as an ultimate effect, improvement of cognitive functions recorded in the NOR test.

## 5. Conclusions

This study provides the first experimental evidence that synaptamide exerts neuroprotective effects in a subchronic MPTP-induced mouse model of PD. Despite the limitations, the present results suggest that synaptamide may influence several key pathological processes in this PD model. These findings support the continued investigation of synaptamide as a potential adjunctive therapeutic agent in PD, while recognizing that further mechanistic and preclinical studies are required before any clinical application can be considered.

## Data Availability

The original contributions presented in this study are included in the article/Supplementary Material. The datasets generated during the current study are available from the corresponding author on reasonable request.

## Supplementary Materials

The following supporting information can be downloaded at: https://www.mdpi.com/article/10.3390/pathophysiology33030042/s1, Figures S4 and S5: Membranes stained by Ponceau S, and the originals of western blots membranes.
