# Supplementary figures and images for: Neuroprotective Potential of Synaptamide in MPTP-Induced Parkinson’s Disease
Source: Pathophysiology. 2026 Jun 25;33(3):42. doi: 10.3390/pathophysiology33030042 (PMC13398039; doi:10.3390/pathophysiology33030042)

**Figure S4**

**IL1 $\beta$**

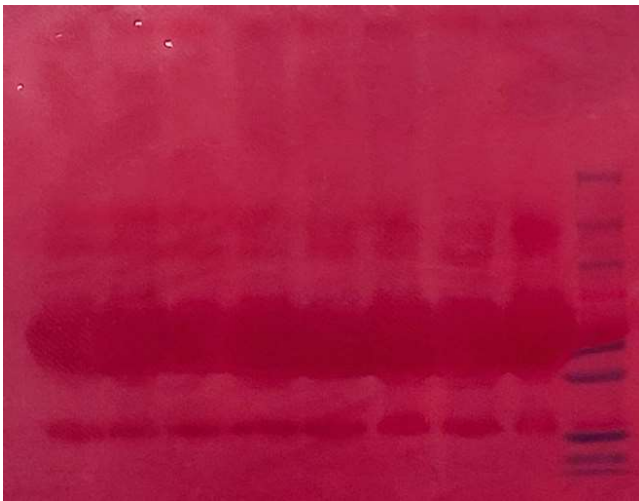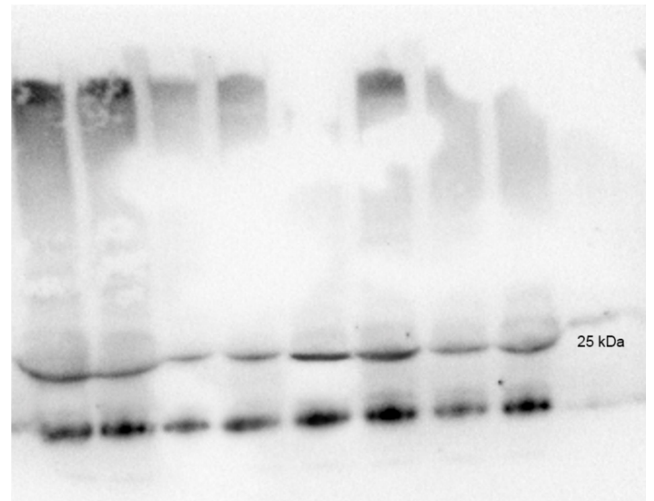

**SNCA**

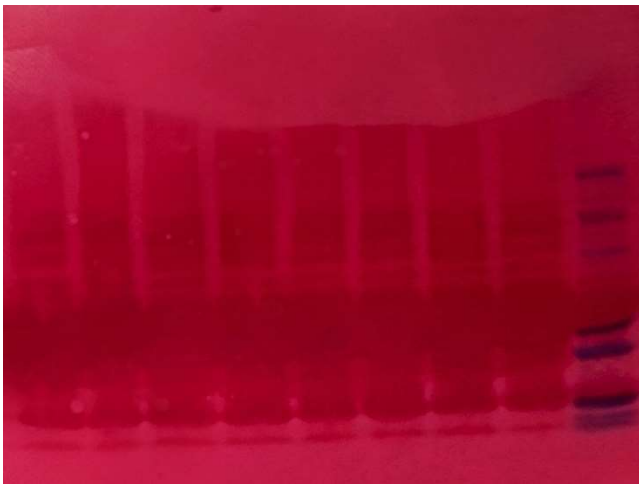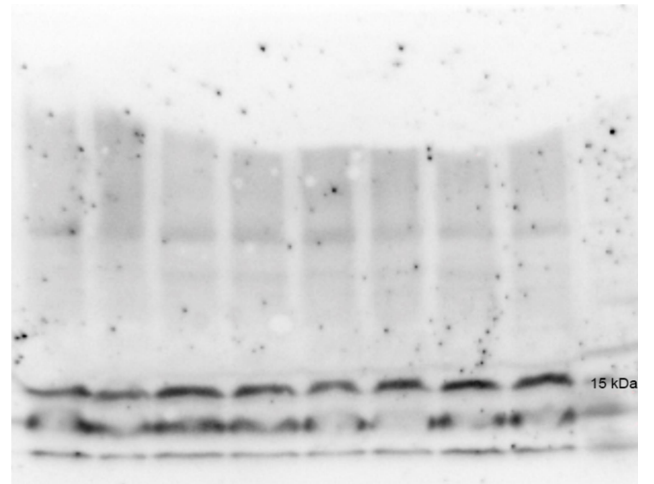

**$\alpha$ -Tubulin**

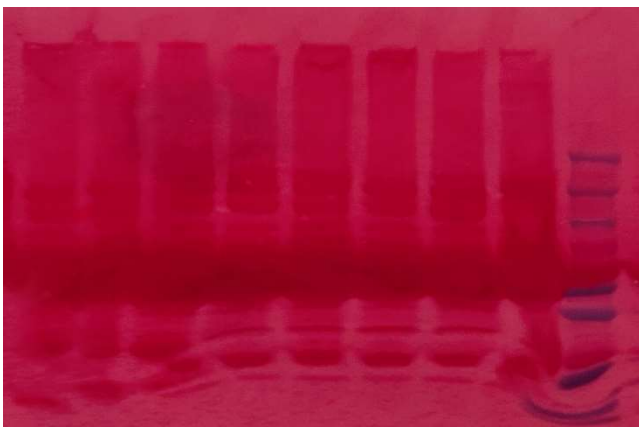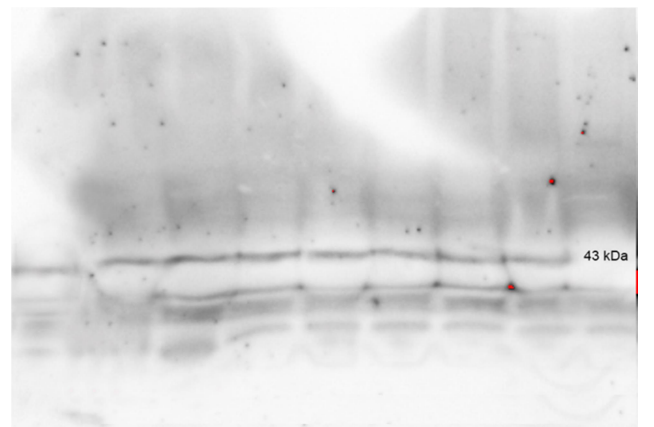

**p-SNCA**

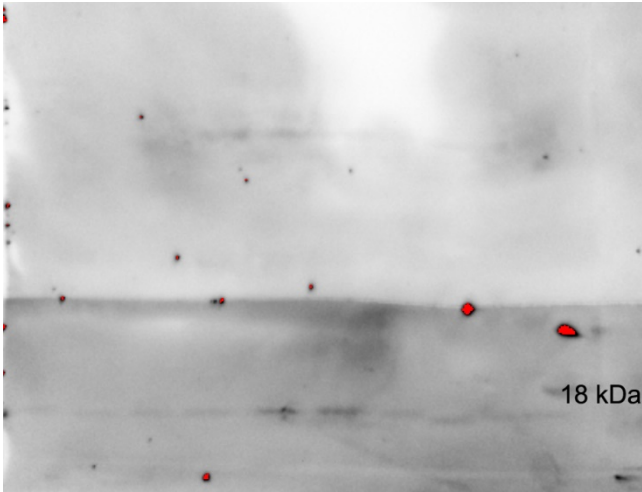

**$\alpha$ -Tubulin**

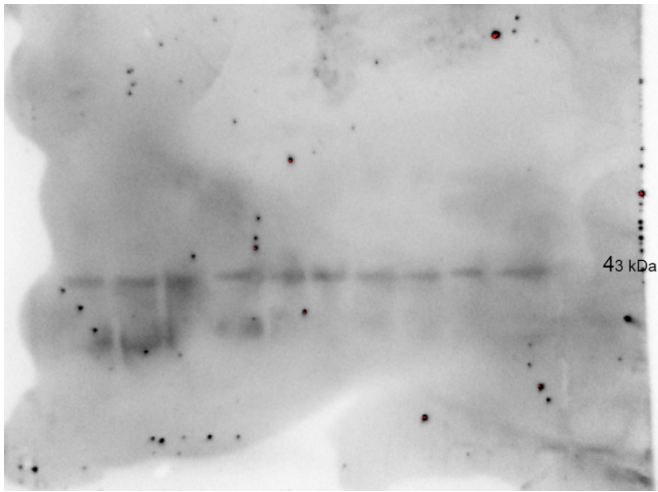

**Figure S5**

**TH**

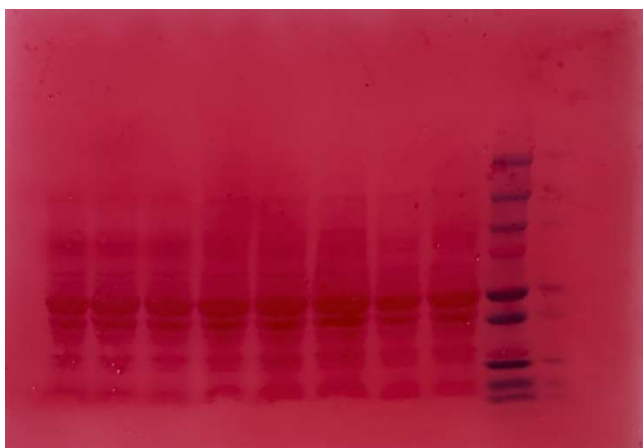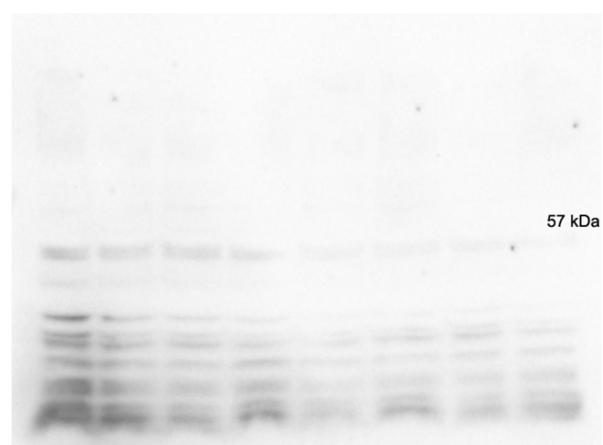

**p-SNCA**

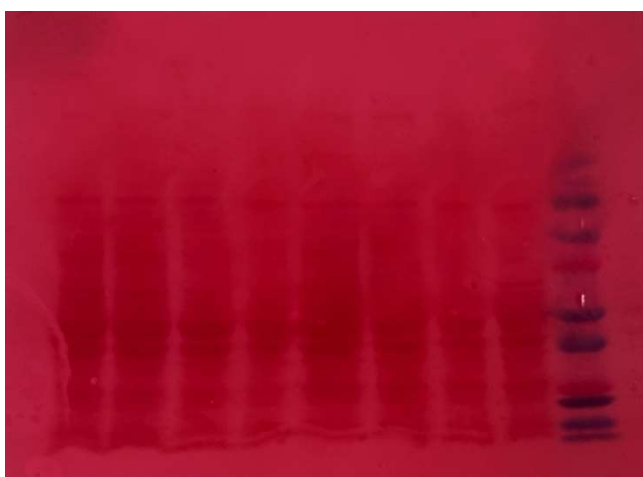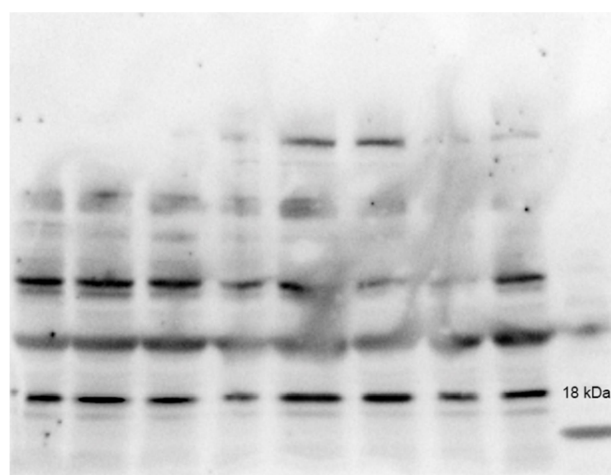

**$\alpha$ -Tubulin**

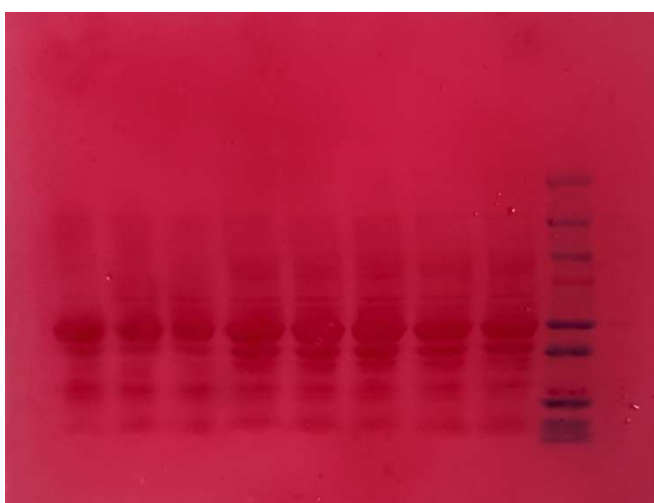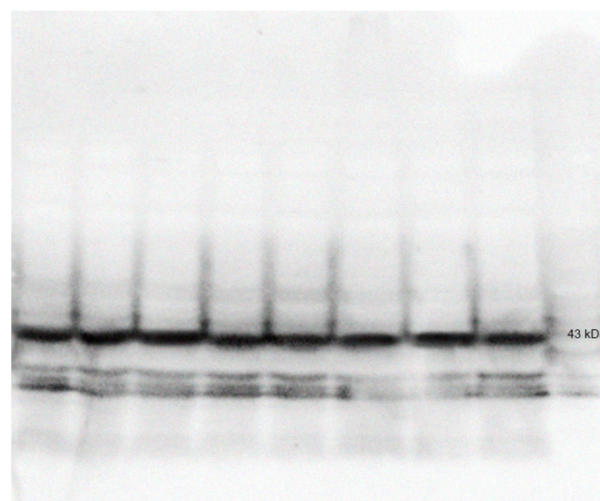

Supplement: Supplementary file 1 [file pathophysiology-33-00042-s001.zip › pathophysiology-4348221-supplementary.pdf]
